# Pyridoxine dependent epilepsies: new therapeutical point of view

**DOI:** 10.1186/s13052-017-0387-3

**Published:** 2017-08-05

**Authors:** Raffaele Falsaperla, Giovanni Corsello

**Affiliations:** 1 Unit of Pediatrics and Pediatric Emergency, University Hospital “Policlinico-Vittorio Emanuele”, Via Plebiscito 628, 95124 Catania, Italy; 2 Department of Maternal and Child Health, University of Palermo, Palermo, Italy

**Keywords:** Pyridoxine dependent epilepsies, Drugs-resistant seizures, Conventional anticonvulsant drugs, Pyridoxine administration, New therapeutical approach

## Abstract

Pyridoxine dependent epilepsies (PDEs) are rare autosomal recessive disorders with onset in neonatal period. Seizures are typically not responsive to conventional antiepileptic drugs, but they cease after parental pyridoxine administration. Atypical forms are characterized partly response to pyridoxine and a late onset of symptoms (up to the age of three years). Prevalence is variable and it has rarely been described. The genes involved in PDEs are the gene encoding for the Alpha-aminoadipic-semialdehyde dehydrogenase (ALDH7A1) and PROSC gene, which encodes a pyridoxal-5-phosphate binding protein. Mutations in the gene encoding for the pyridoxal-5′-phosphate oxidase enzyme (PNPO) are responsible of a clinical entity similar to PDEs responsive to pyridoxal-5-phosphate administration not to pyridoxine administration. PDEs diagnosis is often delayed because they are suspected only after conventional anticonvulsant drugs resistance.

Herein authors aim to present an expert point of view on PDEs in childhood, reviewing the most recent literature data and proposing a new therapeutical approach for seizures of unknown origin in all those children up to the age of three years.

## Main text

Pyridoxine dependent epilepsies (PDEs) are rare autosomal recessive disorders characterized by seizures at neonatal onset not responsive to conventional antiepileptic drugs (CADs), that only cease after parenteral pyridoxine administration They present with seizures in neonatal age, not responsive to conventional antiepileptic drugs, that only cease after parenteral pyridoxine administration. Epidemiology of PDEs has rarely been described, with a considerable heterogeneity of published incidence and prevalence. There are few clinical studies on a cohort basis describing the diagnostic and therapeutic management of PDEs in paediatric age. The disease is usually not suspected before neonatal seizures are considered resistant to conventional anticonvulsant drugs. In regards, there is not a standardized diagnostic work-up for PDEs and this is often the cause for a delayed diagnosis. Conventional antiepileptic drugs are used as first-line treatment. We suggest a new therapeutical approach with intravenous pyridoxine, as first line treatment, in all those children up to the age of three years, with seizures of unknown origin before starting a therapy with conventional antiepileptic drugs.

## Letter to the editor

Pyridoxine dependent epilepsies (PDEs) are rare autosomal recessive disorders characterized by seizures at neonatal onset not responsive to conventional antiepileptic drugs (CADs), that only cease after parenteral pyridoxine administration [1]. Hunt Ad Jr. et al. first described PDEs in 1954.

Atypical forms include seizures partly responsive to pyridoxine, and the late-onset forms, described in children up to the age of three years [1–10].

Diagnostic criteria for this condition include: 1) cessation of clinical and electroencephalographic seizures following iv administration of 100 mg of pyridoxine; 2) no seizure recurrence while on long-term treatment [1]. Laboratory tests, including the dosage of alpha-aminoadipic semihaldeyde (A-AASA), pipecolic acid, pyridoxine and pyridoxal-5′-phosphate oxidase, and DNA studies can be useful to confirm the diagnosis.

PDEs epidemiology has rarely been described, with a considerable heterogeneity; reported birth incidences are 1:20,000 in Germany, 1:396,000 in Netherlands and 1:783,000 in the UK and Ireland [2].

Mutations in the gene encoding for the alpha-aminoadipic-semialdehyde dehydrogenase (ALDH7A1) have been identified at first instance, resulting in the related enzyme deficiency [3]. Though more than 80 gene mutation variants are been associated with PDEs, only 9 represent 61% of PDEs mutations. The most common mutation is the missense p.Glu399Gln in exon 14 representing 30% of all allele variations. The silent mutation p.Val250Val is prevalent in the Caucasian population [6]. There are other clinical entities due to mutations of the gene encoding for the PNPO, on chromosome 17q21.2, with similar clinical features of PDEs, but responsive to pyridoxal-5′-phosphate (PLP) administration, instead of pyridoxine [7]. Recently PROSC gene, which encodes a PLP binding protein, has been identified as responsible of PDEs [8]. Some patients who response to PN or PLP administration don’t have any known mutation of these genes, suggesting that other not known yet genes could be involved in PDEs.

On a clinical point of view, literature data report seizures of various type in PDEs patients, including focal seizures, generalized tonic-clonic seizures, myoclonia, and infantile spasms [9]. However, there are few clinical studies on a cohort basis describing diagnostic and therapeutic management of PDEs in paediatric age. Other reports have been published as single cases. This lack of global vision has caused an under diagnosis of the condition, as the disease is usually suspected after neonatal seizures resistance to CADs. There is not a standardized diagnostic work-up for PDEs and this could cause further diagnostic delay.

The most common CADs used as first-line treatment in this condition are: phenobarbital, benzodiazepine, levetiracetam, valproic acid, and midazolam; other drugs such as vigabatrin, phenytoin, ACTH, and dintoine are less frequently used [1]. The wrong diagnosis of the condition lead to a wrong therapeutic approach, with improper anticonvulsant drugs administration and related adverse events. For this reason, according to literature data, we suggest that an intravenous (IV) pyridoxine test should be performed in all those children up to the age of three years, with seizures of unknown origin, as first line treatment, before starting a therapy with conventional anticonvulsant drugs. In literature a daily dose of 15–30 mg/Kg has been recommended for lifelong treatment, and this should be started after the iv bolus test if positive. If bolus test is negative, we suggest to give both pyridoxine oral therapy for few days, for a possible delayed response, and anticonvulsant therapy. Some patients could need additional anticonvulsant drugs to control seizures [10].

Moreover the oral dose of pyridoxine at 50 mg/Kg twice a day for two days has been found as effective as the iv dose to confirm the diagnosis [4].

Pyridoxine should then be continued as daily treatment, and none of the literature report has described adverse events for this therapy.

It is important identify children with PDEs for prevention in their mothers’ further pregnancies; in fact maternal pyridoxine supplementation during pregnancy is crucial to reduce psychomotor delay in possible affected foetuses.

Further studies are mandatory to determine short- and long-term outcome data for children under pyridoxine treatment and to draft standardized diagnostic and therapeutic protocols.

## Abbreviations

A-AASA: Alpha-aminoadipic semihaldeyde
ALDH7A1: Alpha-aminoadipic-semialdehyde dehydrogenase
CADs: Conventional antiepileptic drugs
IV: Intravenous
PDEs: Pyridoxine dependent epilepsies
PLP: Pyridoxal-5′-phosphate
PNPO: Pyridoxal-5′-phosphate oxidase enzyme
